# Seasonal variation in sex-specific immunity in wild birds

**DOI:** 10.1038/s41598-020-80030-9

**Published:** 2021-01-14

**Authors:** José O. Valdebenito, Naerhulan Halimubieke, Ádám Z. Lendvai, Jordi Figuerola, Götz Eichhorn, Tamás Székely

**Affiliations:** 1 Milner Centre for Evolution, Department of Biology and Biochemistry, University of Bath, Bath, UK; 2 Department of Evolutionary Zoology and Human Biology, University of Debrecen, Debrecen, Hungary; 3 Department of Wetland Ecology, Estación Biológica de Doñana (EBD-CSIC), Seville, Spain; 4 CIBER Epidemiología y Salud Pública (CIBERESP), Seville, Spain; 5 Wildlife Ecology & Conservation Group, Wageningen University & Research, Wageningen, The Netherlands; 6 Vogeltrekstation-Dutch Centre for Avian Migration and Demography (NIOO-KNAW), Wageningen, The Netherlands

**Keywords:** Animal physiology, Behavioural ecology, Immunology

## Abstract

Whilst the immune system often varies seasonally and exhibits differences between males and females, the general patterns in seasonality and sex differences across taxa have remained controversial. Birds are excellent model organisms to assess these patterns, because the immune system of many species is well characterised. We conducted a meta-analysis using 41 wild bird species from 24 avian families to investigate sex differences and seasonal (breeding/non-breeding) variations in immune status, including white blood cell counts, phytohaemagglutinin (PHA) test, bacteria-killing ability (BKA), haemolysis and haemagglutination assays. We found male-biased macrophage concentration, BKA and haemolysis titers, but only during the breeding season. Sex-specific heterophil concentrations, heterophil/lymphocyte ratios and PHA responses differed between breeding and non-breeding, suggesting larger changes in males than in females. Importantly, sex differences in immune status are stronger during the breeding period than during the non-breeding period. Taken together, our study suggests that both seasonal variation and sex differences in immune system are common in birds, although their associations are more complex than previously thought.

## Introduction

To thwart pathogens and keep infections at bay hosts rely on a competent immune system^1^. While the relationship between immune function and individual survival has been well documented^2–4^, there has been relatively little research focused on sex differences in immune defence in free-living animals.

Differences in immune response between the sexes have been described extensively across vertebrates. These sex differences have been traditionally associated with the immunomodulating effect of sex hormones, where oestrogens, found in higher concentrations in females, act as weak immune-enhancers, and androgens, higher in males, as immune-suppressors^5,6^. However, these studies have been centred primarily on humans and laboratory animals, while there is increasing evidence suggesting that the association between sex hormones and sex differences in immunity in the wild are not as simple as first thought. Two independent meta-analysis showed that testosterone did not have a consistent overall immunosuppressive effect in males, and the effect depended on the taxa studied and whether the experimental manipulations involved hormone concentrations above physiological levels^7,8^. A recent study has also challenged the notion of sex biases in immunity by finding no overall sex difference in immune estimates in a large-scale comparative analysis including vertebrates and invertebrates^9^. However, Kelly et al.^9^ showed that some patterns do arise when focusing on specific immune variables and taxonomic groups, such as mammals, which showed a strong male bias in specific pro-inflammatory cytokines. Kelly et al.^9^ did not find overall sexual differences in birds immunity, but they concluded that future studies of sex differences in immunity should include variables known to affect immune functioning, such as age^10^, nutritional state^11^, photoperiod^12^ or seasonality^13^. The latter variable is especially relevant, because seasonal changes, in particular the transition between the non-breeding and the breeding period, involve major physiological and behavioural changes. They may also include pronounced environmental shifts, particularly in species that migrate between breeding and non-breeding grounds, which is the case in many species of birds. Accordingly, several studies have found important sex-specific changes in immunity between the non-breeding and breeding period in birds. For example, Hõrak et al.^14^ found that female Great Tits, *Parus major*, had more circulating lymphocytes than males in spring but not in summer. Merrill et al.^15^ found that male Brown-headed Cowbirds, *Molothrus ater*, showed higher bactericidal capacity than females during the breeding period compared to the non-breeding period. Reasons behind such complex seasonal, species-specific and sex-specific immunity are not fully understood. Recurring explanations include sex-specific energetic and nutritional costs that may be traded off against immunity^16–18^, thus resulting in an impaired immunity in the sex with higher energy expenditure (e.g. courtship displays, egg production, parental care^19–21^).

Alternatively, immune defence may be compromised in situations that cause strain or tension, i.e. stress^22^. Corticosterone, the main circulating glucocorticoid in birds, could play an important role here. First, because corticosterone is involved in regulating the metabolism^23^, and second, as result of an increase in stress-induced corticosterone production (e.g. during territory defence) that could supress immune function^24–26^. However, a comprehensive analysis that simultaneously investigates seasonally-related and sex-specific immunity across bird species is largely lacking. Also, it is unknown whether potential sex-specific or seasonal patterns are consistent between immune parameters^27^.

Here, in order to better understand the variation in avian immune function, we conducted a meta-analysis to test for seasonal (breeding versus non-breeding season) and sexual differences in immunity across bird species. Because of the known effects of ontogeny and captivity on immunity^28,29^, we restricted our analysis to data from free-living adult birds. We included information from nine measurements characterising immune status: the relative frequency of four types of white blood cells (heterophils, lymphocytes, macrophages, eosinophils), the ratio of heterophils/lymphocytes (H/L ratio, a glucocorticoid-mediated immune index of stress), and four widely used immune response indexes (the phytohaemagglutinin test, bacteria-killing ability assay, haemolysis assay, and the haemagglutination assay). For each of these nine immune parameters we estimated their overall meta-analytic means (i.e. estimates of sex-specific immune biases). Based on previous studies^9,30^, we expected no sex difference in white blood cells levels and a small female bias in the immune response indexes. Next, we broke down these overall estimates by season, and computed one estimate for the non-breeding period and one for the breeding period. This allowed us to test if these seasonal estimates were sex-biased, and if season, as a variable, had a significant effect on the immune parameters. Because breeding often incurs increased workload and higher energy demands compared to non-breeding birds in winter^16^, we expected the two periods to differ from each other, and season to significantly affect immune variables^31,32^.

Furthermore, we used the estimates from male and female individuals to test if the sexes could respond differently to the transition between seasons. Males are generally more involved in courting behaviour and intrasexual aggression; therefore, we predicted a possible stress-mediated immunosuppression^26^ in males that could outweigh an alternative immunosuppression due to energetic trade-offs in females^21^. Thus, in the transition from non-breeding to breeding, males may exhibit stronger changes in immune estimates than females.

## Materials and methods

### Literature search

We systematically collected sex-specific white blood cells and immune response data from birds (PRISMA method^33^) using ISI Web of Science (see chart in Fig. S1; list of references in supplementary material). Our inclusion criteria required these data to be: (1) determined from adult birds with known sex, (2) obtained from free-living wild birds (not captive), and (3) from populations that were not experimentally manipulated. In order to conduct the meta-analytic calculations, the selected studies should provide the number of individuals examined per sex, the arithmetic mean of the immune variable measured and an estimate of its variance. We only included publications reporting results for both sexes to avoid difficulties generated by different sampling/diagnostic methods or different populations when calculating individual effect sizes.

### Immune variables

#### White blood cells (WBC)

We used data on the four most abundant WBC circulating in avian blood^34^: heterophils, lymphocytes, macrophages (also known as monocytes), and eosinophils. Basophils counts were discarded because of insufficient data available. The H/L ratio was also collected or calculated using the raw values of heterophils and lymphocytes. Elevated leucocyte number is a symptom of a stress syndrome, inflammatory processes and/or oxidative stress^35^. Usually, leucocytosis is caused by an elevated concentration of heterophils and/or lymphocytes^36,37^. Lymphocytes are immune cells that assist in the recognition and destruction of many types of pathogens. Although sometimes difficult to interpret, decreased lymphocyte concentrations may signal stress-induced immunosuppression^38^, or may indicate a lack of parasite infections^39^. Heterophils are non-specific phagocytic cells that enter the tissues during inflammatory processes. Heterophil concentrations increase as a response to inflammatory processes, stress and infections^37^. Thus, the ratio of these two cell lines is considered a reliable proxy of physiological stress in birds^35,40^. Macrophages and eosinophils are less abundant in the avian blood than lymphocytes and heterophils. Their main function is to phagocytise and present antigens to T lymphocytes (T-cells), and to mediate the defence against parasite infections. Variation in their levels is commonly associated with pathogen infection^34^. WBC data came from apparently healthy animals (i.e. with no obvious signs of disease detected during handling), therefore assumed to represent baseline levels. The time between capture and sampling was not always available (details in Table S1), and Davis^41^ showed that within one hour of capture the total leucocyte counts decreased as a result of handling stress, whereas proportions of each leucocyte type did not differ significantly. Therefore, we calculated WBC proportions (from the total number of leucocytes) to reduce between-study variation.

#### Estimates of immune response

We used four widely accepted measures of immune response in birds: the (1) phytohaemagglutinin test (PHA), that consists of a subcutaneous injection of this mitogen (phytohaemagglutinin) that triggers a local immune response mediated mostly by T-cell infiltration. Components of the innate and adaptive immune system take part in the response, which is estimated by measuring the degree of swelling of the skin, usually 24 h post-injection^42^. The (2) bacteria-killing ability assay (BKA) quantifies the ability of proteins in the plasma (such as complement, natural antibodies, and lysozymes) and/or phagocytic cells to kill bacteria^43^. The (3) haemolysis and (4) haemagglutination assays use foreign red blood cells (usually rabbit) to quantify titres of complement-like lytic enzymes (i.e. lysis, HL) and non-specific natural antibodies (i.e. agglutination, HA) in plasma^44^. From each study we recorded whether the study was done during the breeding or the non-breeding season (hereafter season). Details of the breeding status extracted from each study are presented in Tables S1 and S2.

We used standard deviation (SD) as estimate of variance. When standard error was provided, we calculated SD using Eq. (1):

1 $$SD = SE \times \sqrt n$$

where $SE$ is the standard error, and $n$ is the sample size.

When 95% confidence intervals were given (in two studies), SD was calculated with Eq. (2):

2 $$SD = \frac{{\sqrt n \times \left( {upper CI {-}lower CI} \right)}}{2\delta }$$

where $n$ is the sample size, $CI$ the confident intervals, and $\delta$ is the value for the *t*-distribution with degrees of freedom equal to the sample size minus 1 and a probability of 0.05^45^.

### Statistical analysis

#### Phylogenetic meta-analysis

To investigate sex biases in immunity, a phylogenetic multilevel meta-analysis was performed using the R package ‘metafor’^46^. Effect sizes were computed using Hedge’s *g* for standardised means because of its common use in ecology literature and for including a correction for small sample sizes^47,48^. Effect sizes are the standardised mean difference between two groups, which in our case corresponded to the mean of males relative to the female mean. Negative values of *g* indicate a female bias in the immune parameter studied and positive values a male bias. We conducted multilevel random-effect meta-analyses using the previously computed effect sizes as response variable and season (non-breeding/breeding) as moderator (i.e. fixed-effect). Phylogeny (a variance–covariance matrix) and study (to account for more than one species and/or immune estimate per study) were added as random-effect variables. We used the avian phylogeny proposed by Jetz et al.^49^ and the analyses were conducted using consensus trees (one for each type of immune variable, Fig. S2) obtained by 50% majority-rule^50,51^ from 1000 randomly selected trees from a pool of 10,000 available trees (http://birdtree.org) using the methodology described by Rubolini et al.^52^. These phylogenetic trees were not fully resolved, and polytomies were arbitrarily resolved by adding a branch distance of 10^–8^ to one randomly chosen branch in the polytomy using the function ‘multi2di’ from the R package ‘ape’^53^. Publication bias (due to missing studies that were not published because of negative or null results^54^) was evaluated by inspecting the symmetry in funnel plots and using the Egger’s regression test^55,56^ by including the standard error of the effect sizes as an additional moderator within the model. If the intercept significantly deviated from zero (significance of *p* < 0.10^55^), the overall relationship between the precision and size of studies included in the data set was considered asymmetrical or, in other words, biased^56^. Of the nine fitted models, only macrophages and eosinophils suggested presence of publication bias (both *p* < 0.001). Diagnostic tests for identifying influential data points and outliers, and rules for excluding these types of cases are not well established, particularly for multivariate/multilevel meta-analytical models^57^. We used the approach described by Habeck and Schultz^58^ by identifying the influential outliers causing the bias and running the models after excluding these values. We report results after removing one effect size from the final model of macrophages, and two from the model of eosinophils (see Table S3 for the final sample sizes used in the analyses). The effect of season on the immune sex-bias was tested using the Omnibus test (*QM*) for moderators (a Wald-type Chi-squared) implemented within the function ‘rma.mv’ (metafor R package), which tests whether the explained heterogeneity by a parameter (here, season) is significantly greater than the unexplained overall heterogeneity^46^. The HL and HA assays were excluded from further analysis because only estimates of breeding birds were available. We used Cochran’s *Q* test to estimate whether the (residual) heterogeneity among effect sizes was greater than expected by sampling error alone^59^. We also calculated the variance in effect sizes due to phylogenetic relatedness (*I*^2^_phylogeny_), differences among studies (*I*^2^_study_), and the total variance attributed to the random effect variables (i.e. the addition of the two effects, *I*^2^_total_).

#### Generalised linear mixed models

To explore if seasonal changes affected the sexes independently, we fitted generalised linear mixed models by Markov chain Monte Carlo techniques using the R package ‘MCMCglmm’^60^. This analysis differs from the previous in that here we analysed variation of each sex parameters according to season, instead of one ‘combined’ effect size. This approach helps to understand how each sex responds to season, because changes in effect size estimates from the non-breeding period to the breeding period may be the result of increases or reduction in one or both sexes at once. Each of these seven models (HA and HL were excluded) had immune variables as response variable, and season, sex (females/males), and the two-way interaction of season and sex as explanatory (fixed-effect) variables. All models included study and phylogeny as random-effect variables. The H/L ratio was log-transformed. The H/L ratio and PHA models were run with a Gaussian family distribution. The rest of the models were run using a binomial family distribution. To investigate whether the above comparisons may have been confounded by different species composition in the breeding and non-breeding samples, we ran these models two times. First using the full dataset, and then using a subset of the data that included only those species for which we had data from both non-breeding and breeding seasons (Table S4). We used parameter expanded (random-effects) and inverse-Wishart priors (fixed-effects) based on improving model convergence. Further details of model specification are given in the supplementary material. Convergence and autocorrelation levels were assessed through the Gelman-Rubin test^61^, trace graphs and the ‘autocorr’ function, implemented in the R package ‘coda’^62^. MCMCglmm results are expressed as posterior mean, lower and upper 95% credible intervals, and significance as a pMCMC value.

## Results

### Sex biases in immunity and the effect of season (meta-analysis)

**Figure 1:**
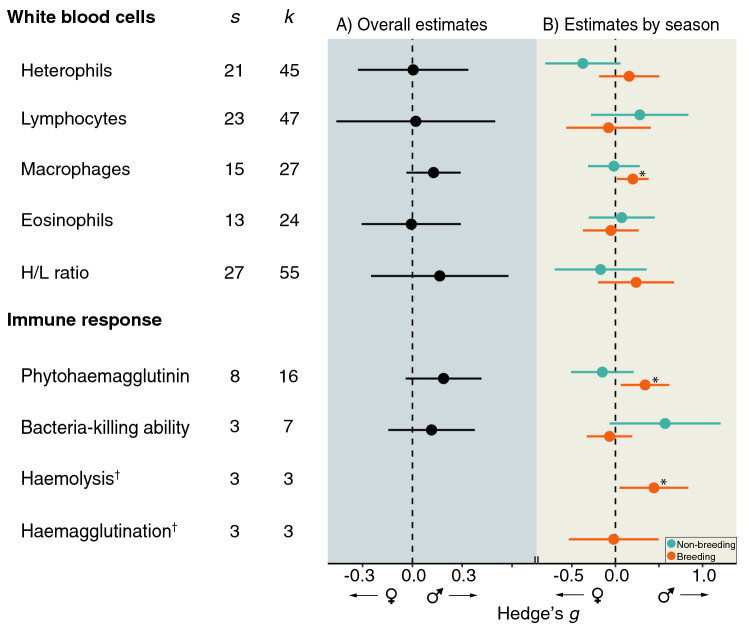
Sex bias in white blood cells and immune response assays in adult wild birds (weighted average effect sizes and 95% confidence intervals). (**A**) Overall in immune estimates. (**B**) Immune estimates for non-breeding (in cyan) and breeding (in orange) birds. Weighted averages were tested whether they differed significantly from zero (i.e. no sex bias, dashed line; see statistics in Table 1), where positive estimates mean male bias and negative female bias. *s*, number of species; *k*, number of effect sizes; H/L ratio, heterophils/lymphocytes ratio; *statistical significance (*p* < 0.05); ^†^data from breeding birds only.

Our results show that across all immune variables, while there was no overall difference between males and females (Fig. 1A), there was an important variation in sex differences between the non-breeding and the breeding period (Fig. 1B; Table 1). Macrophage concentration, haemolysis score and PHA response were significantly male-biased during breeding (Fig. 1B). During the non-breeding period, BKA tended to be higher in males (*p* = 0.089) while heterophil concentration tended to be higher in females (*p* = 0.079). Both phylogeny and study explained an important proportion of the variance in immune variables (Table 1).

**Table 1:** Sex bias in (a) white blood cell types and the H/L ratio, and (b) immune response assays in adult wild birds. *p *values < 0.05 in bold.

Immune variable	*I*^2^_phylogeny_ (%)	*I*^2^_study_ (%)	*I*^2^_total_ (%)	*Q*_REML_ (*P*)	Overall estimates		Estimates by season			
					Overall (95% CI)	*Z* statistic (*P*)	Non-breeding (95% CI)	*Z* statistic (*P*)	Breeding (95% CI)	*Z* statistic (*P*)
**(a) White blood cells**										
Heterophils	23.69	46.08	69.76	171.238 (< 0.001)	0.005 (− 0.327, 0.337)	0.027 (0.978)	− 0.373 (− 0.804, 0.057)	− 1.698 (0.089)	0.158 (− 0.186, 0.502)	0.902 (0.367)
Lymphocytes	45.17	33.91	79.07	182.957 (< 0.001)	0.020 (− 0.457, 0.498)	0.084 (0.933)	0.280 (− 0.279, 0.839)	0.981 (0.327)	− 0.079 (− 0.564, 0.406)	− 0.318 (0.750)
Macrophages	< 0.01	22.98	22.98	26.780 (0.367)	0.128 (− 0.036, 0.291)	1.531 (0.126)	− 0.018 (− 0.314, 0.279)	− 0.117 (0.907)	0.200 (0.020, 0.380)	**2.175 (0.030)**
Eosinophils	38.52	0.00	38.52	26.520 (0.277)	− 0.007 (− 0.305, 0.292)	− 0.045 (0.964)	0.073 (− 0.307, 0.452)	0.375 (0.708)	− 0.052 (− 0.371, 0.268)	− 0.317 (0.752)
H/L ratio	40.18	33.39	73.58	191.669 (< 0.001)	0.143 (− 0.296, 0.582)	0.639 (0.523)	− 0.171 (− 0.700, 0.358)	− 0.634 (0.526)	0.240 (− 0.199, 0.680)	1.071 (0.284)
**(b) Immune response**										
PHA	0.00	9.54	9.54	13.839 (0.462)	0.188 (− 0.040, 0.415)	1.614 (0.107)	− 0.150 (− 0.508, 0.208)	− 0.821 (0.412)	0.341 (0.063, 0.619)	**2.407 (0.016)**
BKA	< 0.01	0.00	< 0.01	15.122 (0.010)	0.115 (− 0.145, 0.376)	0.868 (0.385)	0.571 (− 0.066, 1.207)	1.758 (0.079)	− 0.067 (− 0.328, 0.194)	− 0.503 (0.615)
HL	33.39	0.00	33.39	2.080 (0.354)	–	–	–	–	0.443 (0.048, 0.837)	**2.199 (0.028)**
HA	28.45	33.48	61.93	4.605 (0.100)	–	–	–	–	− 0.019 (− 0.533, 0.495)	− 0.074 (0.941)

H/L ratio, heterophils/lymphocytes ratio; PHA, phytohaemagglutinin test; BKA, bacteria-killing ability assay; HL, haemolysis assay; HA, haemagglutination assay; *I*^2^_phylogeny_, variance due to phylogenetic relatedness; *I*^2^_study_, variance due to differences among studies; *I*^2^_total_, total variance attributed to the random effect; *Q*_REML_, Cochran’s *Q* test for (residual) heterogeneity. *Z* statistic tests if immune parameter estimate differ from zero (no sex difference).

Seasonal changes had a significant effect on the sex bias estimates of three immune parameters: heterophil concentration, H/L ratio and PHA response (Omnibus test of coefficients [df = 1]: 8.131, *p* = 0.004; 8.547, *p* = 0.003; 4.832, *p* = 0.028, respectively; Table 2). These results indicate that, in these immune parameters, the immune estimates from the non-breeding and breeding periods were significantly different from each other. In all cases the direction of the skew was towards males. A non-significant trend in the opposite direction was found for lymphocyte concentration and for BKA, where estimates obtained in the breeding season deviated towards females (Table 2).

**Table 2:** Omnibus test of coefficients (*QM*) testing for the effect of season on the sex bias of the immune parameters studied. *p *values < 0.05 in bold.

Immune variable	*QM* (df = 1)	*P*
**White blood cells**		
Heterophils	8.131	**0.004**
Lymphocytes	3.453	0.063
Macrophages	1.662	0.197
Eosinophils	0.488	0.485
H/L ratio	8.547	**0.018**
**Immune response**		
PHA	4.832	**0.028**
BKA	3.301	0.069

H/L ratio, heterophils/lymphocytes ratio; PHA, phytohaemagglutinin test; BKA, bacteria-killing ability assay.

### Effect of seasonal changes on males and females (GLMM analysis)

**Figure 2:**
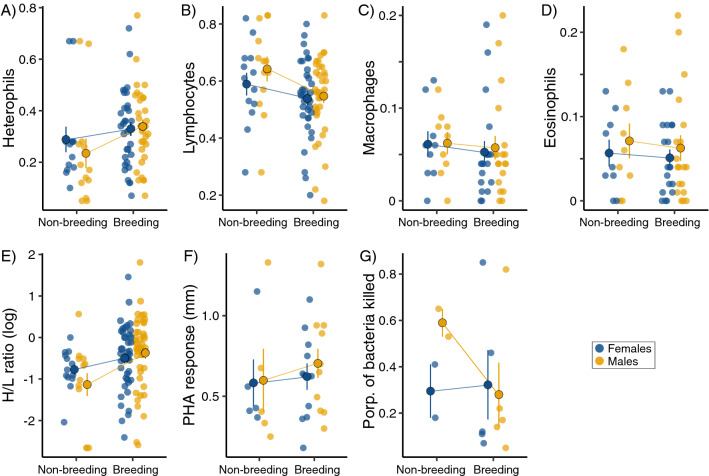
Characteristics of the immune system in wild birds. White blood cells (**A**–**D**, expressed as the proportion of the total white blood cell count), heterophils/lymphocytes (H/L) ratio (**E**), phytohaemagglutinin response (**F**), and bacteria-killing ability (**G**) in breeding and non-breeding birds (blue and yellow dots refer to females and males, respectively). Large outlined dots and whiskers are arithmetic means and standard errors, respectively.

The GLMM–MCMC models revealed a significant interaction between season and sex for heterophil concentration and H/L ratio, indicating that these variables show a greater change between non-breeding and breeding season in males than in females (Fig. 2A,E; Table 3). These results were consistent between models using the whole data set and those using a subset of species for which data during both the non-breeding and breeding season were available (Table S5). Also, for BKA, seasonal changes tended to differ between males and females when tested with the full data set (*p* = 0.078), but the pattern became weaker when using the subset of data (Table S5), arguably due to low sample size in this variable (Fig. 2G). The other immune parameters (lymphocytes, macrophages, eosinophils, PHA) showed no significant sex differences in the change between non-breeding and the breeding period, suggesting that males and females either increase or decrease their levels in comparable proportions (Fig. 2, Table 3).

**Table 3:** White blood cell levels and immune response in wild birds in relation to sex and season (MCMC generalised linear mixed models; *n*, total number of individuals; *s*, number of species). *p *values < 0.05 in bold.

		95% credibility intervals		
	Post. mean	Lower	Upper	*P*
**(a) Heterophils (n = 90, s = 21)**				
Intercept	− 0.448	− 1.336	0.338	0.236
Season (breeding)^a^	− 0.113	− 0.579	0.317	0.602
Sex (males)^b^	− 0.396	− 0.723	− 0.124	**0.012**
Season (breeding)^a^ * sex (males)^b^	0.429	0.036	0.760	**0.018**
Random				
Study	0.560	0.070	1.069	
Phylogeny	0.411	< 0.001	1.310	
Residual	0.081	0.033	0.136	
**(b) Lymphocytes (n = 94, s = 23)**				
Intercept	− 0.091	− 0.768	0.548	0.782
Season (breeding)^a^	0.035	− 0.405	0.393	0.864
Sex (males)^b^	0.254	− 0.026	0.580	0.094
Season (breeding)^a^ * sex (males)^b^	− 0.221	− 0.570	0.148	0.238
Random				
Study	0.170	< 0.001	0.426	
Phylogeny	0.336	< 0.001	0.840	
Residual	0.109	0.052	0.163	
**(c) Macrophages (n = 56, s = 15)**				
Intercept	− 3.494	− 5.220	− 1.857	**0.002**
Season (breeding)^a^	− 0.476	− 1.026	0.151	0.112
Sex (males)^b^	0.019	− 0.375	0.411	0.932
Season (breeding)^a^ * sex (males)^b^	0.071	− 0.485	0.494	0.750
Random				
Study	1.065	< 0.001	3.393	
Phylogeny	1.839	< 0.001	5.211	
Residual	0.008	< 0.001	0.029	
**(d) Eosinophils (n = 56, s = 13)**				
Intercept	− 3.873	− 5.978	− 2.199	**0.002**
Season (breeding)^a^	0.220	− 0.425	0.793	0.448
Sex (males)^b^	0.251	− 0.187	0.741	0.256
Season (breeding)^a^ * sex (males)^b^	− 0.042	− 0.620	0.491	0.878
Random				
Study	1.483	0.140	3.671	
Phylogeny	2.548	0.422	5.853	
Residual	0.043	< 0.001	0.134	
**(e) H/L ratio (n = 110, s = 27)**				
Intercept	− 0.577	− 1.200	0.096	0.088
Season (breeding)^a^	0.054	− 0.277	0.422	0.764
Sex (males)^b^	− 0.361	− 0.677	− 0.006	**0.032**
Season (breeding)^a^ * sex (males)^b^	0.483	0.095	0.875	**0.014**
Random				
Study	0.517	0.137	0.973	
Phylogeny	0.233	< 0.001	0.777	
Residual	0.182	0.130	0.237	
**(f) PHA response (n = 32, s = 8)**				
Intercept	0.648	0.182	1.139	**0.012**
Season (breeding)^a^	0.097	− 0.140	0.320	0.420
Sex (males)^b^	0.017	− 0.219	0.240	0.884
Season (breeding)^a^ * sex (males)^b^	0.060	− 0.239	0.309	0.664
Random				
Study	0.050	< 0.001	0.231	
Phylogeny	0.155	< 0.001	0.424	
Residual	0.034	0.018	0.056	
**(g) BKA assay (n = 14, s = 3)**				
Intercept	− 0.253	− 4.987	4.873	0.864
Season (breeding)^a^	− 0.195	− 1.709	1.318	0.718
Sex (males)^b^	1.356	0.011	2.889	0.056
Season (breeding)^a^ * sex (males)^b^	− 1.594	− 3.499	0.219	0.078
Random				
Study	6.441	< 0.001	22.95	
Phylogeny	5.375	< 0.001	20.10	
Residual	0.598	0.066	1.510	

^a^Relative to the non-breeding period.

^b^Relative to females.

## Discussion

To our knowledge, this is the first multi-species analysis investigating the effect of seasonal variation on sex-specific immunity in wild birds. We showed an overall lack of sex differences in the immune variables studied. However, when taking season into account, subtle but consistent patterns arise indicating that males are undergoing more substantial reorganization of their leukocyte composition during reproduction than females.

Similar to Kelly et al.^9^, the overall meta-analysis of the immune parameters showed no significant sex biases in immunity, although with subtle variations of male and female biases in the estimates. In multilevel meta-analysis, non-significant results could originate from small effect sizes being close to zero (i.e. no sex difference). However, the heterogeneity attributed to random effect variables was rather high (*I*^*2*^ and *Q* test^63^), suggesting that our data set had great variation of opposing effect sizes (i.e. some species estimates showing a male bias and others a female bias). Breaking the immune estimates down by season revealed notable sex differences between the non-breeding and breeding period, with macrophage concentration, PHA response and haemolysis score being male-biased, and a significant seasonal influence on the estimated sex bias for heterophil concentration, H/L ratio and PHA response. Heterophils and lymphocytes make up to 95% of the total leucocyte count^64^. Both cell types have important roles in innate immunity, but only lymphocytes participate in adaptive immunity^34,36^. Macrophage levels were male-biased during the breeding period, but no sex differences were found for levels of eosinophils. Macrophages and eosinophils are specialised against unspecific cells like apoptotic cells or microbes, and against parasite infections, respectively^1^. Studies reporting sex differences in these two leucocyte lines in birds are scarce. Variation in levels of eosinophils are attributed to different levels of infection by gastrointestinal parasites in birds^65,66^, and sex differences in macrophage gene expression associated to the sex chromosomes have been reported in chicken^67^.

Seasonally varying levels in (1) stress (defined as a physiological response due to strain or tension), (2) hormones and (3) workload may form the basis of mechanisms that could explain our findings. First, stressors associated with breeding could cause immunosuppression. It has been suggested that behaviours such as sexual display and nestling feeding in birds are comparable to strenuous exercise in that they impose a high metabolic rate^68,69^. In addition, because males are in general more aggressive and dominant than females, in periods of low food abundance (such as winter) males could secure their access to food over females, which seems to cause strain in birds^70–72^. This could explain our results for H/L ratio, since increments in H/L ratio appear to be associated with sustained stress in birds^35,40,73^. Although the H/L ratio was not sex-biased during the non-breeding or the breeding season, both estimates were different from each other, and males experienced a greater change between the seasons than females. Second, the breeding period in birds is characterised by behavioural changes triggered by the sex hormones. Androgens and oestrogens have traditionally been thought to influence immunity in birds by up- or down-regulating their immune system. However, current evidence disregards sex hormones (mostly testosterone) as important immune modulators in birds^7,8^. For instance, Roberts et al.^74^ found no effect of testosterone on immune response in Japanese Quail, *Coturnix japonica*. Li et al.^75^ found that in Eurasian Tree Sparrow, *Passer montanus*, testosterone concentration was positively correlated with the strength of PHA response in males, whereas in females the correlation was negative. Additionally, Duffy et al.^76^ concluded that the increase in plasma corticosterone upon treatment with testosterone implants in European Starlings, *Sturnus vulgaris*, was the likely cause of immunosuppression in males and females rather than testosterone itself. Conclusions from studies in wild birds have been based mainly on correlational observations, which may obscure the real effect of sex hormones on immunity. Furthermore, our results are consistent with previous literature failing to find consistent support for the immunocompetence-handicap hypothesis^7,9,77,78^.

Third, reproduction requires temporarily elevated energy and nutrient input, which could compromise immune function^16,79,80^. Trade-offs between reproduction and self-maintenance may vary both between the sexes and over specific stages of reproduction while each sex invests in traits that will maximise reproductive success^81^. Accordingly, but depending on breeding system and sex roles, during mating it might be the males but during egg production and incubation the females that compromise their immune function relatively more. For example, in a clutch size manipulation experiment in Common Eiders, *Somateria mollissima*, Hanssen et al.^82^ showed that females incubating larger clutches lost more body mass and showed reduced immune function (lymphocyte levels and specific antibody response). While in lekking males of Greater Sage-grouse, *Centrocercus urophasianus*, alfa males showed a daily energy expenditure two times higher than a non-displaying male and four times higher than their basal metabolic rate^68^. Unfortunately, the data collected for our meta-analysis were obtained from studies that sampled at various moments throughout the entire breeding period and from species with different breeding systems, which prevented us from drawing further conclusions. Likewise, the present analysis relied on a selection of more generic indicators of (innate) immunity, and future research will profit by including also more specific indicators and those that belong to the adaptive arm of the immune system. Moreover, immune tolerance and autoimmunity can significantly influence the cost balance and, therefore, the outcome of reproduction-immunity trade-offs^83,84^.

Although data on immune response variables were not available for many species, we did find differences between males and females. The four immune assays analysed reflect innate immunity, except the PHA test that, if repeated more than once, also includes components of the adaptive immunity^85^. The PHA test and the haemolysis assay were significantly male-biased during breeding, although the latter estimate was obtained only from three effect sizes. Generally, the PHA response in birds appears to decrease during breeding^42,86^, although no association with breeding was found in Chinstrap Penguins, *Pygoscelis antarctica*^27^. In Eurasian Tree Sparrow, Li et al.^75^ found no differences in PHA responses between breeding males and females, while Zhao et al.^87^ found that body condition but not breeding stage correlated with their haemolysis levels. Interestingly, in our analysis the PHA test and the BKA assay showed opposite responses to season (Figs. 1B and 2F,G). In both cases the differences seemed to be largely driven by changes in males (Fig. 2F,G). However, with a relatively small sample size and considering the subset analysis, the results of the model interaction of BKA assay should be taken cautiously. Yet another possible explanation for our results on immune response variables might be based on sexual selection theory, and predicts that the competing sex (males in most mating systems) will evolve higher innate immune response. According to this scenario, selection would favour strong inflammation responses as an aid for healing wounds, because the competing sex is more involved in aggressive interactions causing physical injury^88,89^. The inclusion of mating system should thus be considered in future studies in order to test this hypothesis.

Here we have shown that across wild birds, sex differences in certain measures of immune status and response associated to the breeding season may occur. The exact causes of these seasonal patterns of sexual changes in immune function are difficult to identify. In addition to the complex nature of the avian immune system, a number of unaccounted variables could directly or indirectly confound our analysis, such as genetic, environmental and ecological factors (like photoperiod or mate competition), with the potential of affecting one or several immune components, and in different sex-specific fashion. The scarcity of available studies to date prevented us also from exploring factors like mating system and parental care, which seem important to further understand the causes of seasonal and sexual changes in immunity. Nonetheless, our results highlight sexual differences in immune function as a relevant topic that requires further attention in wild birds.

## Supplementary Information

Supplementary information.

## Data Availability

The full dataset and R code can be found at 10.6084/m9.figshare.13476819.v1.
