# Supplementary material for: Seasonal variation in sex-specific immunity in wild birds
Source: Sci Rep. 2021 Jan 14;11:1349. doi: 10.1038/s41598-020-80030-9 (PMC7809460; doi:10.1038/s41598-020-80030-9)
Supplement: Supplementary file 1 — Supplementary information. [file 41598_2020_80030_MOESM1_ESM.pdf]

Seasonal variation in sex-specific immunity in wild birds

José O. Valdebenito[1]*, Naerhulan Halimubieke[1], Ádám Z. Lendvai[2], Jordi Figuerola[3,4], Götz Eichhorn[5,6] & Tamás Székely[1,2]

[1]Milner Centre for Evolution, Department of Biology and Biochemistry, University of Bath, Bath, United Kingdom

[2]Department of Evolutionary Zoology and Human Biology, University of Debrecen, Debrecen, Hungary

[3]Department of Wetland Ecology, Estación Biológica de Doñana (EBD-CSIC), Seville, Spain

[4]CIBER Epidemiología y Salud Pública (CIBERESP), Seville, Spain

[5]Wildlife Ecology & Conservation Group, Wageningen University & Research, Wageningen, The Netherlands

[6]Vogeltrekstation-Dutch Centre for Avian Migration and Demography (NIOO-KNAW), Wageningen, the Netherlands

*Correspondence: José O. Valdebenito, e-mail: jov23@bath.ac.uk

Supplementary material

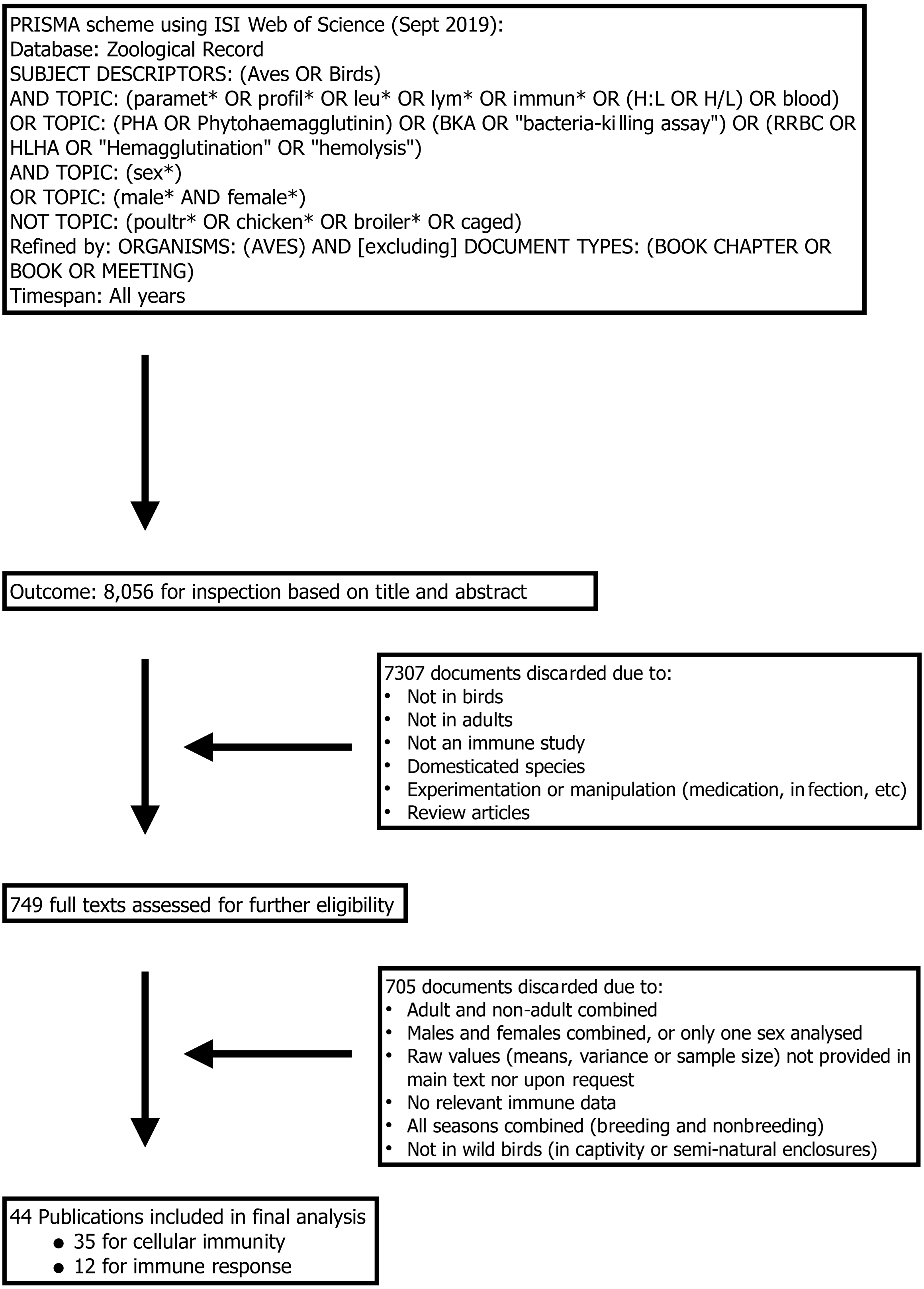

Figure S1. Searching parameters and PRISMA scheme followed to obtain publications used in analysis.

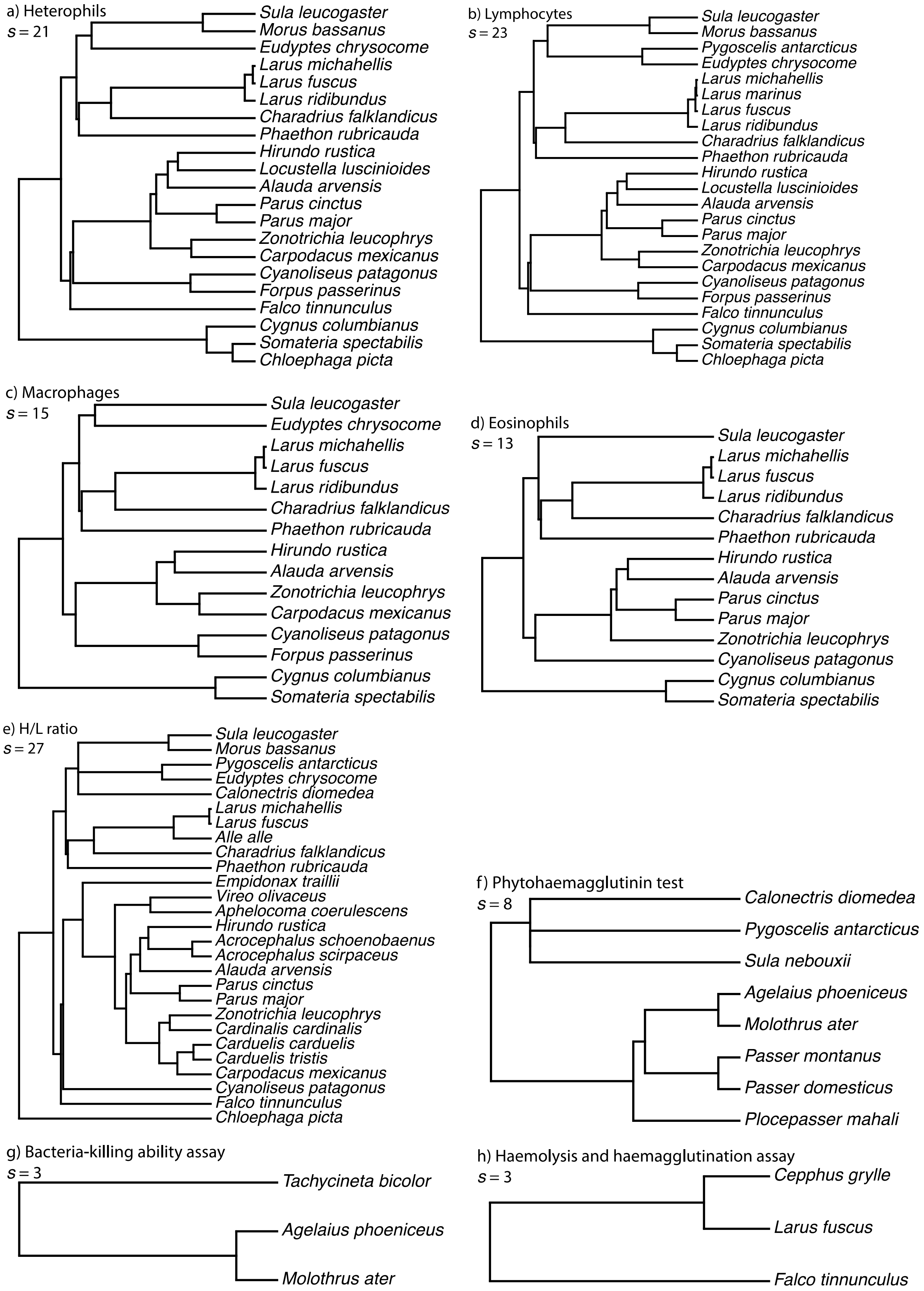

Figure S2. Phylogenetic trees used in meta-analyses and MCMC generalised linear mixed models. H/L ratio = heterophils/lymphocytes ratio. *s* refers to number of species.

Reference list of studies included in the meta-analysis

Cellular immunity (WBC)

1. Arriero et al. 2015. Variation in Immune Parameters and Disease Prevalence among Lesser Black-Backed Gulls (Larus fuscus sp.) with Different Migratory Strategies. PloS One 10: e0118279
2. Bonier et al. 2006. Sex-specific consequences of life in the city. Behavioral Ecology 18: 121‚Äì129
3. Boughton et al. 2006. An introduced generalist parasite, the sticktight flea Echidnophaga gallinacea, and its pathology in the threatened florida scrub-jay Aphelocoma coerulescens. J. Parasitol. 92(5): 941‚Äì948
4. Brousseau-Fournier et al. 2014. Immunologic and genotoxic profile of Northern Gannet (morus bassanus) from Bonaventure Island. Journal of Xenobiotics 4: 53-55
5. Camplani et al. 1999. Carotenoids, sexual signals and immune function in barn swallows from Chernobyl Proc. R. Soc. Lond. B 266: 1111-1116
6. Carbo-Ramirez & Zuria. 2017. Leukocyte profile and body condition of the house finch in tro sites with different levels of ubanization in central Mexico. Ornitologia Neotropical 28: 1-10
7. D'amico et al. 2017. Physiologic parameters and their response to hadling stress in a neotropical migratory shorebird during the nonbreeding season. Journal of Wildlife Diseases 53(3): 1-10
8. Dehnhard & Hennicke. 2013. Leucocyte profiles and body condition in breeding brown boobies and red-tailed tropicbirds, effects of breeding stage and sex. Australian Journal of Zoology 61: 178-185
9. Dehnhard et al. 2011. Do leucocyte profiles reflect temporal and sexual variation in body condition over the breeding cycles in southern rockhopper penguins? J Ornithol 152:759-768
10. Garcia et al. 2010. Does breeding status influence haematology and blood biochemistry of yellow-legged gulls? Acta Biologica Hungarica 61(4): 391-400
11. Giudici et al. 2010. Physiological ecology of breeders and sabbaticals in a pelagic seabird. Journal of Experimental Marine Biology and Ecology 389: 13-17
12. Gladbach et al. 2010. Variations in leucocyte profiles and plasma biochemistry are related to different aspects of parental investment in male and female Upland geese Chloephaga picta leucoptera. Comparative Biochemistry and Physiology, Part A 156: 269-277
13. Granthon & Williams. 2017. Avian malaria body condition and blood parameters in four songbirds. The Wilson Journal of Ornithology 129(3): 492-508.
14. Hõrak et al. 1998. Health and reproduction, the sex-specific clinical profile of great tits Parus major in relation to breeding. Can. J. Zool. 76: 2235-2244

15. Hegemann et al. Frontiers in Zoology (2013) 10: 77, & Oecologia (2012) 170: 605-618[1]
16. Jakubas et al. 2013. Factors Affecting Haematological Variables and Body Mass of Reed Warblers (Acrocephalus scirpaceus) and Sedge Warblers (A. schoenobaenus). Annales Zoologici Fennici 50(3): 146-157
17. Kelly et al. 2012. Carotenoid-based ornaments of female and male american goldfinches Spinus tristis show sex-specific correlations with immune function and metabolic rate. Physiological and Biochemical Zoology 85(4): 348-363
18. Krama et al. 2013. Physiological condition and blood parasites of breeding Great Tits: a comparison of core and northernmost populations. J Ornithol 154: 1019-1028
19. Krams et al. 2010. Effects of forest management on haematological parameters, blood parasites, and reproductive success of the Siberian tit (Poecile cinctus) in northern Finland. Ann. Zool. Fennici 47: 335-346
20. Krams et al. 2011. Extremely low ambient temperature affects haematological parameters and body condition in wintering Great Tits (Parus major). J Ornithol 152: 889-895
21. Kristiansen et al. 2006. Carotenoid coloration in great black-backed gull Larus marinus reflects individual quiality. J. Avian Biol. 37: 6-12
22. Kulaszewicz et al. 2015. Variation of the Savi's warbler Locustella luscinoides leucocyte profiles and body condition in relation to age, sex and moult. Ann. Zool. Finici 52: 01-14
23. Lopez et al. 2008. Carotenoid based masks in the european goldfinch carduelis carduelis reflects different information in males ad females. Ardea 96(2): 233-242
24. Maney et al. 2008. Carotenoid-based plumage coloration predicts leukocyte parameters during the breeding season in northern Cardinals. Ethology 114: 369-380
25. Milani et al. 2012. Hematology, Plasma Chemistry, and Bacteriology of Wild Tundra Swans (Cygnus columbianus) in Alaska. Journal of Wildlife Diseases 48(1): 212-215
26. Moreno et al. 1998. Breeding time, health and immune response in the chinstrap penguin Pygoscelis antarctica. Oecologia 115 :312-319
27. Munoz & De la Fuente. 2003. Lymphoid distribution in the migratory gull Larus rudibundus. Comparative Biochemistry and Physiology Part A 136: 749-756
28. Owen et al. 2005. Habitat and sex differences in ohysiological condition of breeding southwestern willow flycatchers Empidonax traillii extimus. The Auk 122(4): 1261-1270
29. Palacios et al. 2018. Cellular and humoral immunity in two highly demanding energetic life stages: reproduction and moulting in the Chinstrap Penguin. J Ornithol 159: 283,Äì290.

[1]The authors provided a dataset that was the source of the two publications cited.

30. Pap. 2002. Breeding time and sex-specific health status in the barn swallow Hirundo rustica. Can. J. Zool. 80: 2090-2099
31. Parejo & Silva. 2009. Immunity and fitness in a wild population of Eurasian kestrels Falco tinnunculus. Naturwissenschaften 96: 1193-1202
32. Plischke et al. 2010. Leucocytes In Adult Burrowing Parrots Cyanoliseus patagonicus in the wild, variation between contrasting breeding seasons, gender and individual condition. J Ornithol 151:347-354
33. Scott et al. 2010. Health Evaluation of Western Arctic King Eiders (Somateria spectabilis). Journal of Wildlife Diseases 46(4): 1290-1294
34. Sheridan et al. 2004. Weak association between measures of health and reproductive success in green-rumped parrotlets (Forpus passerinus) in Venezuela. The Auk 121(3): 717-725
35. Wojczulanis-Jakubas et al. 2015. A big storm in a small body, seasonal changes in body mass, hormone concetrations and leukocyte profile in the little auk Alle alle. Polar Biol 38: 1203-1212

Immune response tests and assays

1. Arriero et al. 2015. Variation in Immune Parameters and Disease Prevalence among Lesser Black-Backed Gulls (Larus fuscus sp.) with Different Migratory Strategies. PloS One 10: e0118279
2. Berzins et al. 2011. Sex-Specific Effects of Increased Incubation Demand on Innate Immunity in Black Guillemots. Physiological & Biochemical Zoology 84: 222-229
3. Cram et al. 2015. Immune Response in a Wild Bird Is Predicted by Oxidative Status, but Does Not Cause Oxidative Stress. PloS One 10: e0122421
4. Forero et al. 2006. Ecological and physiological variance in T-cell mediated immune response in Cory's Shearwaters. Condor 108: 865-876
5. Houdek et al. 2011. Innate Immunity is Not Related to the Sex of Adult Tree Swallows During the Nestling Period. Condor 113: 853-859
6. Li et al. 2015. Changes in phytohaemagglutinin skin-swelling responses during the breeding season in a multi-brooded species, the Eurasian Tree Sparrow: do males with higher testosterone levels show stronger immune responses? Journal of Ornithology 156: 133-141
7. Merrill et al. 2013. Immune Function in an Avian Brood Parasite and Its Nonparasitic Relative. Physiological and Biochemical Zoology 86: 61-72
8. Navarro et al. 2007. Digit ratios (2D:4D), secondary sexual characters and cell-mediated immunity in house sparrows Passer domesticus. Behavioral Ecology & Sociobiology 61: 1161-1168

9. Palacios et al. 2018. Cellular and humoral immunity in two highly demanding energetic life stages: reproduction and moulting in the Chinstrap Penguin. J Ornithol 159: 283–290.
10. Parejo & Silva. 2009. Immunity and fitness in a wild population of Eurasian kestrels Falco tinnunculus. Naturwissenschaften 96: 1193-1202
11. Santiago-Quezada et al. 2015. Secondary phytohaemagglutinin (PHA) swelling response is a good indicator of T-cell-mediated immunity in free-living birds. Ibis 157: 767-773
12. Stewart & Merrill. 2015. Host sex and parasitism in Red-winged Blackbirds (Agelaius phoeniceus): examining potential causes of infection biases in a sexually dimorphic species. Can. J. Zool. 93: 21-29

Table S1. Breeding status and time from capture to blood sampling in studies investigating cellular immunity (WBC).

Reference number	Species	Breeding status	Breeding status	Stated capture-sampling time
1	*Larus fuscus*	Breeding	Captured at nest during breeding season	Not specified
2	*Zonotrichia leucophrys*	Breeding	Captured pairs with mist nets near the nest during breeding season. Breeding status confirmed with behavioural observations	Within 3 mins after capture
3	*Aphelocoma coerulescens*	Breeding	Not specified; sampled during the breeding season. Inferred by capture dateΨ	Within 1 min after capture
4	*Morus bassanus*	Breeding	Captured at nest during breeding season	Immediately after capture
5	*Hirundo rustica*	Breeding	Captured at the gate of breeding colony (in stables)	Not specified
6	*Haemorhous mexicanus*	Breeding	Not specified; sampled during the breeding season. Inferred by capture dateΨ	Immediately after capture
7	*Charadrius falklandicus*	Non-breeding	Captured with cannon net to non-breeding birds outside the breeding season	10–232 min, mean = 105.2 (SD = 56.7)
8	*Phaethon rubricauda*<br>*Sula leucogaster*	Breeding	Captured at nest during incubation and chick rearing	Within 3–10 mins after capture
9	*Eudyptes chrysocome chrysocome*	Breeding	Captured during incubation	Within 3 mins after capture
10	*Larus michahellis*	Breeding<br>Non-breeding	Captured at nest for breeders<br>Captured outside the breeding season for non-breeders	Not specified
11	*Calonectris diomedea*	Breeding<br>Non-breeding	Captured at breeding colony<br>Divided in breeders and sabbaticals (non-breeders)	Not specified
12	*Chloephaga picta leucoptera*	Breeding	Captured during chick rearing with whoosh nets	Not specified
13	*Vireo olivaceus*	Breeding	Captured by mist-netting during the breeding season	Within 30 mins after capture
14	*Parus major*	Breeding	Captured from nest boxes during the breeding season	Not specified; stated: “at capture”

15	*Alauda arvensis*	Breeding<br>Non-breeding	Captured breeding birds 2006–2009<br>captured birds at moulting, autumn migration and wintering 2007–2008	Within 2–35 min, median = 5 min
16	*Acrocephalus scirpaceus*<br>*Acrocephalus schoenobaenus*	Breeding<br>Non-breeding	Captured at different times of the year. The last one corresponding to post-breeding, dispersal and start of autumn migration	Not specified
17	*Spinus tristis*	Breeding	Mist nets by feeders in breeding grounds. Birds were determined as breeders based on bill and plumage colours	Within 60 mins after capture
18	*Parus major*	Breeding	Captured at nest boxes during breeding	Not specified; stated: "at capture"
19	*Poecile cinctus*	Breeding	Captured at nest boxes during breeding	Not specified
20	*Parus major*	Non-breeding	Captured with mist nets by feeders in winter	Within 1 min after capture
21	*Larus marinus*	Breeding	Captured at nest in breeding colony	Not specified; handling time and lymphocyte levels not correlated P = 0.34
22	*Locustella luscinoides*	Non-breeding	Captured with mist nest post breeding and beginning of dispersal and migration	Not specified
23	*Carduelis carduelis*	Breeding	Captured during breeding season. Presence of brood patch or cloacal protuberance	Not specified
24	*Cardinalis cardinalis*	Breeding	Captured during breeding season. Presence of brood patch or cloacal protuberance	Not specified
25	*Cygnus columbianus*	Non-breeding	Captured during moulting in the breeding grounds, previous start of migration	Not specified
26	*Pygoscelis antarcticus*	Breeding	Captured at nest in breeding colony	Not specified
27	*Larus ridibundus*	Non-breeding	Captured in non-breeding grounds (Spain)	Not specified
28	*Empidonax traillii extimus*	Breeding	Captured during breeding season. Presence of brood patch or cloacal protuberance	Not specified
29	*Pygoscelis antarcticus*	Breeding<br>Non-breeding	Captured by hand at the nest during nesting for breeders and later at moulting for non-breeders	Not specified
30	*Hirundo rustica*	Breeding	Captured using mist nets when arriving to breeding site and then during check rearing	Not specified
31	*Falco tinnunculus*	Breeding	Captured at nest boxes during breeding season	Not specified; stated: "at capture"

32	*Cyanoliseus patagonus*	Breeding	Captured at nests on cliffs during breeding season	Within 30 mins after capture
33	*Somateria spectabilis*	Breeding	Captured at the onset of nesting during breeding season	Not specified
34	*Forpus passerinus*	Breeding	Captured at nest boxes or using mist nets close to nests	Not specified
35	*Alle alle*	Breeding	Captured at nest in the colony (stated that only took breeding adults)	Within 3 mins after capture

Ψ Species breeding period was assumed according to del Hoyo et al. (2019) Handbook of the Birds of the World Alive. Lynx Edicions, Barcelona.

Table S2. Breeding status in studies investigating immune function.

Reference number	Species	Breeding status	Breeding status
1	*Larus fuscus*	Breeding	Captured at nest during breeding season
2	*Cepphus grylle*	Breeding	Captured when leaving nest in burrows in breeding colonies
3	*Plocepasser mahali*	Non-breeding	Captured at nest during breeding season; breeders were discarded after behavioural observations
4	*Calonectris diomedea*	Breeding	Captured by hand on their nests during the incubation, in colony
5	*Tachycineta bicolor*	Breeding	Captured from nest boxes during breeding season
6	*Passer montanus*	Breeding	Captured with mist nets and breeding status determined by anatomy and behaviour
7	*Molothrus ater*<br>*Agelaius phoeniceus*	Breeding<br>Non-breeding	Captured during the breeding season; only using older birds (according to plumage) to ensure they got a mate<br>Recaptured outside the breeding season
8	*Passer domesticus*	Non-breeding	Captured just before start of breeding season. Reproductive status inferred by date of captureΨ
9	*Pygoscelis antarcticus*	Breeding<br>Non-breeding	Captured by hand at the nest during nesting for breeders and later at moulting for non-breeders
10	*Falco tinnunculus*	Breeding	Captured at nest boxes during breeding season
11	*Sula nebouxii*	Breeding	Captured at nest in breeding colony
12	*Agelaius phoeniceus*	Breeding	Captured during breeding season while territory formation till fledging of chicks

Ψ Species breeding period was assumed according to del Hoyo et al. (2019) Handbook of the Birds of the World Alive. Lynx Edicions, Barcelona.

Table S3. Number of studies, species and effect sizes used in present analyses.

Immune variable	Studies	Species	Effect sizes
White blood cells			
Heterophils	23	21	45
Lymphocytes	25	23	47
Macrophages	14	15	27
Eosinophils	12	13	24
H/L ratio	27	27	55
Immune response			
PHA	8	8	16
BKA	3	3	7
Haemolysis	3	3	3
Haemagglutination	3	3	3

H/L ratio = heterophils/lymphocytes ratio, PHA = phytohaemagglutinin test, BKA = bacteria-killing ability assay.

Model specification of Markov chain Monte Carlo simulations for generalised linear mixed models

Apart from the H/L ratio and the PHA, all our variables were proportions. Proportions were considered as success/failure (cbind(success, failure)) and the H/L ratio was log-transformed. The H/L ratio and PHA models were run with a Gaussian family distribution, whereas the rest of the models were run using a binomial family distribution (specified as 'multinomial2'). We used parameter expanded priors for the random effects (V = diag(1), nu = 1, alpha.mu = c(0), alpha.V = diag(1)), inverse Wishart priors (V = 1, n = 0.002) for the residuals and normal distributions centred on zero with large variances as fixed effects priors (default prior in function *MCMCglmm*). These priors were chosen to improve model convergence while being minimally informative (random effects) or completely uninformative (fixed effects). The models of macrophages in both full data set and subset analysis were run across 25,000,000 iterations with a thin of 20,000 and a burn-in of 5,000,000. The rest of the models were run across 1,603,000 iterations, thin of 1,600 and a burn-in of 3000. In all seven models, the potential scale reduction factor from the Gelman-Rubin test was 1.04 or lower, which is below the threshold of 1.1 indicating model convergence. Autocorrelation was also low, always below the threshold of 0.1.

Table S4. Number of species and number of individuals included in MCMC generalised linear mixed models of the full data set, and in the subset of data including species for which there were data from both the non-breeding and breeding periods.

	Analysis using full data set		Analysis using subset of data	
	Number of species	Number of individuals	Number of species	Number of individuals
Heterophils	21	90	3	28
Lymphocytes	23	94	4	30
Macrophages	15	56	2	14
Eosinophils	13	56	3	18
H/L ratio	27	110	7	48
PHA	8	32	5	24
BKA	3	14	2	12

Table S5. Sex difference and seasonal variation in white blood cell counts and immune responses in wild bird species for which there were data from both the non-breeding and breeding periods (MCMC generalised linear mixed models; *n* = total number of individuals; *P*-values < 0.05 highlighted in bold).

		95% credibility intervals		
	Post. mean	Lower	Upper	*P*
a) Heterophils (*n* = 28)				
Intercept	-1.008	-2.261	0.424	0.124
Season (breeding)[a]	0.030	-0.630	0.676	0.946
Sex (males)[b]	-0.649	-1.145	-0.242	**0.004**
Season (breeding)[a]*sex (males)[b]	0.572	-0.052	1.213	0.084
Random				
Study	0.550	< 0.001	1.852	
Phylogeny	0.592	< 0.001	2.395	
Residual	0.123	0.014	0.265	
b) Lymphocytes (*n* = 30)				
Intercept	-0.048	-1.717	1.330	0.980
Season (breeding)[a]	-0.005	-0.563	0.556	0.994
Sex (males)[b]	0.334	-0.021	0.755	0.088
Season (breeding)[a]*sex (males)[b]	-0.280	-0.834	0.283	0.352
Random				
Study	0.260	< 0.001	0.983	
Phylogeny	1.186	< 0.001	3.588	
Residual	0.119	0.029	0.231	
c) Macrophages (*n* = 14)				
Intercept	-2.679	-5.357	0.317	**0.050**
Season (breeding)[a]	-0.598	-1.538	0.223	0.142
Sex (males)[b]	0.025	-0.501	0.496	0.930
Season (breeding)[a]*sex (males)[b]	0.241	-0.943	1.325	0.674
Random				

Study	1.786	< 0.001	7.264	
Phylogeny	2.287	< 0.001	10.91	
Residual	0.035	< 0.001	0.145	
c) Eosinophils (*n* = 18)				
Intercept	-2.933	-4.824	-1.205	**0.018**
Season (breeding)[a]	0.410	-0.277	1.076	0.224
Sex (males)[b]	0.265	-0.292	0.838	0.346
Season (breeding)[a]*sex (males)[b]	-0.382	-1.278	0.411	0.376
Random				
Study	0.850	< 0.001	3.622	
Phylogeny	1.138	< 0.001	4.594	
Residual	0.05768	< 0.001	0.207	
c) H/L ratio (*n* = 48)				
Intercept	-0.553	-1.353	0.183	0.120
Season (breeding)[a]	0.066	-0.342	0.492	0.744
Sex (males)[b]	-0.389	-0.750	-0.015	**0.040**
Season (breeding)[a]*sex (males)[b]	0.556	0.091	1.070	**0.040**
Random				
Study	0.394	0.021	0.989	
Phylogeny	0.177	< 0.001	0.709	
Residual	0.212	0.126	0.306	
d) PHA response (*n* = 24)				
Intercept	0.805	-0.281	1.984	0.104
Season (breeding)[a]	-0.094	-0.334	0.153	0.422
Sex (males)[b]	0.091	-0.076	0.273	0.282
Season (breeding)[a]*sex (males)[b]	-0.070	-0.397	0.214	0.654
Random				
Study	0.224	< 0.001	1.021	
Phylogeny	0.531	< 0.001	1.881	
Residual	0.030	0.013	0.054	
d) BKA assay (*n* = 12)				
Intercept	-1.711	-6.111	2.292	0.234
Season (breeding)[a]	0.364	-1.213	1.989	0.624
Sex (males)[b]	-0.215	-1.414	1.110	0.702
Season (breeding)[a]*sex (males)[b]	1.501	-0.450	3.736	0.144
Random				
Study	3.328	< 0.001	14.14	
Phylogeny	3.707	< 0.001	11.33	
Residual	0.671	0.067	1.62	

[a]Relative to the non-breeding period

[b]Relative to females
